# Associations of posttraumatic stress disorder symptoms with amyloid burden in cognitively normal older adults

**DOI:** 10.3389/fnagi.2024.1422862

**Published:** 2024-12-03

**Authors:** Lei Zhang, Yi-Miao Gong, San-Wang Wang, Pei-Ling Shi, Ming-Zhe Li, Xin Wen, Di-Xin Wang, Yong-Bo Zheng, Yong Han

**Affiliations:** ^1^Department of Nutrition,Henan Mental Hospital, The Second Affiliated Hospital of Xinxiang Medical University, Xinxiang, China; ^2^Peking University Sixth Hospital, Peking University Institute of Mental Health, NHC Key Laboratory of Mental Health (Peking University), National Clinical Research Center for Mental Disorders (Peking University Sixth Hospital), Chinese Academy of Medical Sciences Research Unit (No. 2018RU006), Peking University, Beijing, China; ^3^Peking-Tsinghua Center for Life Sciences and PKU-IDG/McGovern Institute for Brain Research, Peking University, Beijing, China; ^4^Department of Psychiatry, Renmin Hospital of Wuhan University, Wuhan, China; ^5^Department of Psychiatry, Henan Mental Hospital, The Second Affiliated Hospital of Xinxiang Medical University, Xinxiang, China; ^6^Henan Collaborative Innovation Center of Prevention and Treatment of Mental Disorder, Xinxiang, China; ^7^Henan Key Lab of Biological Psychiatry, International Joint Research Laboratory for Psychiatry and Neuroscience of Henan, Xinxiang Medical University, Xinxiang, China; ^8^Key Laboratory of Brain Health Intelligent Evaluation and Intervention, Ministry of Education (Beijing Institute of Technology), Beijing, China

**Keywords:** amyloid, posttraumatic stress disorder symptom, older adult, cross-sectional study, dementia

## Abstract

**Background:**

Posttraumatic stress disorder (PTSD) is associated with the development of dementia. However, the link between PTSD and preclinical Alzheimer’s disease pathology (amyloid *β* [Aβ]) remains controversial. Moreover, the correlation between the severity of PTSD with Aβ levels remains unknown.

**Methods:**

This cross-sectional study sought to investigate the associations of PTSD symptoms with global and regional brain Aβ burden. To this end, data were obtained from participants in the Anti-Amyloid Treatment in Asymptomatic Alzheimer’s Disease (A4) Study. In addition, we explored the association between the severity of PTSD symptoms and Aβ levels.

**Results:**

A total of 4,228 participants aged 65 to 85 years were included in the final analysis. The results showed that PTSD symptoms were significantly associated with higher global Aβ levels (1.15 ± 0.20 vs. 1.09 ± 0.19; β = 0.056; *p* < 0.001), after adjusting for covariates. The association between PTSD symptoms and Aβ levels was not affected by sex, age, *ApoE* genotype, or psychiatric diseases. Similarly, PTSD symptoms were significantly associated with Aβ levels in all subregions, including the anterior cingulate, posterior cingulate, parietal cortex, precuneus, temporal cortex, and frontal cortex. In addition, the group with severe PTSD symptoms (1.22 ± 0.24) exhibited higher global Aβ levels than the groups with moderate (1.14 ± 0.19) or mild (1.12 ± 0.20) symptoms or the control (1.08 ± 0.18), with *p* < 0.001.

**Conclusion:**

The findings imply a close relationship between PTSD and brain Aβ levels, irrespective of sex, age, *ApoE* genotype, or psychiatric diseases. More well-designed studies are needed to further explore the relationship and mechanism underlying the association between PTSD and Aβ burden.

## Introduction

Posttraumatic stress disorder (PTSD) is one of the most common psychopathological consequences of exposure to trauma such as physical and sexual assaults, fatal accidents, catastrophic pandemics, or other extremely stressful events (Shalev et al., 2017; Yuan et al., 2021). Symptoms of PTSD may include flashbacks, nightmares, and severe anxiety, as well as uncontrollable thoughts about the event (Brewin et al., 2017). The lifetime prevalence of PTSD varies according to social background and country of residence, ranging from 1.3 to 12.2%, and the 1-year prevalence is 0.2 to 3.8% (Karam et al., 2014). In addition, PTSD imposes a great burden on individuals and society, and studies suggest that PTSD is associated with multiple negative health outcomes, including disability, chronic illnesses, and even suicide (Rajkumar, 2023).

In addition to the above negative health outcomes, suggestive effects have been demonstrated for an association between PTSD and the development of later-life negative cognitive outcomes, including worse-than-normal changes with aging and Alzheimer’s disease (Greenberg et al., 2014). A longitudinal cohort study of approximately 500,000 civilians found that PTSD was associated with an increased risk of dementia over an average of 8 years of follow-up (Flatt et al., 2018). In addition, increasing evidence has identified PTSD as a possible risk factor for dementia in military veterans (Rafferty et al., 2018). In general, veterans with PTSD were found to exhibit an approximately 2-fold increased risk of dementia compared to those without PTSD (Mawanda et al., 2017; Qureshi et al., 2010; Yaffe et al., 2010). These epidemiological studies imply that PTSD is associated with a high risk of dementia (Flatt et al., 2018; Rafferty et al., 2018) or AD (Greenberg et al., 2014).

Because there are no available treatments for dementia and AD, the identification of potential risk factors may reduce the risk of developing dementia and help ease the personal and economic burden caused by dementia (Livingston et al., 2024; Zheng et al., 2021). As a biomarker for dementia, detection of the amyloid burden in cognitively normal older adults has clinical implications for the prevention and treatment of cognitive decline and dementia in their early stages, and it is of primary significance to make efforts to avoid the accumulation of Aβ in the brain (Raza et al., 2021; Zheng et al., 2020, 2022). Despite multiple epidemiological studies finding that PTSD is associated with a high risk of dementia or AD, the majority of research focused on PSTD and Aβ has shown no significant association between these two factors (Elias et al., 2020; Weiner et al., 2022; Weiner et al., 2017). For example, a study found that PTSD did not significantly increase the risk for AD, as measured by amyloid PET, in veterans using the Alzheimer’s Disease Neuroimaging Initiative (Weiner et al., 2017). A cross-sectional study also indicated that there was no association between AD pathology, including Aβ and tau, and PTSD of up to 50 years duration (Elias et al., 2020). Similarly, a null association between PTSD and Aβ or tau accumulation was validated in veterans without dementia (Weiner et al., 2022). These inconsistent findings suggest the necessity for further investigation of the link between PSTD symptoms and Aβ levels.

The association between PTSD symptoms and regional Aβ levels, as well as the relationship between the severity of PTSD symptoms and amyloid burden, has also raised concerns in recent years. A study pointed out that there was no association of PTSD symptoms with Aβ levels in the subregions of the brain, including the frontal, cingulate, parietal, and temporal regions in veterans (Marcolini et al., 2023). With regard to the relationship between the severity of PTSD symptoms and Aβ burden, Caprioglio et al. found that in participants with subjective cognitive decline, amyloid positivity was associated with a higher risk of PTSD symptoms scores but no probable presence of severe PTSD (Caprioglio et al., 2023). This limited evidence further indicates the importance of investigating the relationship between PTSD symptoms and regional Aβ burden as well as between the severity of PTSD symptoms and Aβ burden. Furthermore, the findings on the association between PTSD and Aβ burden are inconclusive, and evidence on the link between PTSD and Aβ burden in cognitively normal older adults is lacking.

Based on these considerations, we aimed to identify a clear association of PTSD symptoms with global and regional Aβ burden; moreover, the link between the severity of PTSD symptoms and Aβ burden was also investigated. In this study, we analyzed a sample of 4,228 cognitively unimpaired older adults with self-reported PTSD symptoms, health information, and amyloid PET imaging. We hypothesized that (1) PTSD symptoms are associated with increased Aβ deposition in the whole-brain and regional brain areas, including frontal, temporal, parietal, precuneus, anterior cingulate, and posterior cingulate and (2) the severity of PSTD symptoms is associated with increased Aβ levels.

## Materials and methods

This cross-sectional study was conducted between April 2014 and December 2017. Participants screened for inclusion in the A4 Study were included in this study if they had undergone an 18F-florbetapir PET scan, had *ApoE* genotype information, completed a battery of neuropsychological testing, scored between 25 and 30 on the Mini-Mental State Examination (MMSE), had a Clinical Dementia Rating of 0, and were aged between 65 and 85 years. Exclusion criteria for the A4 Study have been described previously (Sperling et al., 2014; Zheng et al., 2023). Briefly, participants were excluded from the A4 Study if they were taking a prescription Alzheimer’s medication or had a current serious or unstable illness that could interfere with the study. This study was approved by the institutional review boards of all participating institutions, and written informed consent was obtained from all participants. This study followed the Strengthening the Reporting of Observational Studies in Epidemiology (STROBE) reporting guidelines (Insel et al., 2021). The study also received approval from the Institutional Review Board of the Peking University Sixth Hospital for the use of A4 data.

### PTSD symptoms assessment

The Impact of Event Scale (IES) is widely used to assess PTSD, with satisfactory test–retest reliability (r = 0.79 to 0.89) and internal consistency (Cronbach’s *α* = 0.78 to 0.82) (Horowitz et al., 1979; Joseph, 2000). The IES had 15 items and took 5–10 min to complete. Respondents were asked to rate the frequency, on a 4-point scale, with which each symptom has occurred over the last week. The 4 points were: 0 (not at all), 1 (rarely), 3 (sometimes), and 5 (often). The total scores ranged from 0 to 75, and the interpretation of the total score was based on the following dimensions of posttraumatic stress symptoms: 0 to 8 as subclinical range, 9 to 25 as mild range, 26 to 43 as moderate range, and 44+ as severe range. Participants were considered to have PTSD symptoms if the score exceeded the cutoff point of 26, based on the established literature (Horowitz et al., 1979; Sterling, 2008).

### ^18^F-Florbetapir PET imaging

Florbetapir PET scan images were acquired in accordance with previous studies (Clark et al., 2012; Joshi et al., 2015). A 10-min PET acquisition was performed approximately 50 min after administration of 370 MBq (10 mCi) florbetapir. Images were acquired with a 128 × 128 matrix and reconstructed with iterative or row action maximization likelihood algorithms with a post-reconstruction Gaussian filter. Images for each subject were converted to Montreal Neurologic Institute (MNI) standard stereotactic brain atlas space using a specialized F 18 Florbetapir PET template (Joshi et al., 2015). The resulting transformation matrix was then used to spatially normalize all PET image frames for each subject to the MNI space. The SUVR values for all scans were calculated using the whole cerebellum as a reference. Aβ positivity was defined as an 18F-florbetapir PET SUVR ≥1.10, while Aβ negativity was defined as an SUVR <1.10, as previously described (Clark et al., 2012; Joshi et al., 2012).

Semiautomated quantitative analysis was performed using the mean signal of six predefined anatomically relevant cortical regions of interest (frontal, temporal, parietal, precuneus, anterior cingulate, and posterior cingulate), with the whole cerebellum as a reference region (Clark et al., 2012).

### Covariates

Covariates included demographic information (age, sex, body mass index [BMI], marital status, and educational attainment); habits (sleep duration, alcohol consumption, and cigarette consumption); *ApoE* genotypes; and comorbidities (psychiatric, neurological, cardiovascular, and endocrinological diseases). A summary of the covariates is provided in Supplementary Table S1. There were six *ApoE* genotypes: ε22, ε23, ε24, ε33, ε34, and ε44. The most common *ApoE* allele, *ApoE*-ε3, was associated with an average risk of AD, whereas the *ApoE*-ε4 and *ApoE*-ε2 alleles were linked with higher and lower AD risks, respectively (Liu et al., 2013; Shen and Jia, 2016).

### Statistical analysis

Descriptive statistics were used to present the demographic and clinical characteristics of participants according to their PTSD status. Independent sample t-tests and χ2 tests were used to compare continuous and categorical variables, respectively. Multivariate linear regression analyses were performed to explore the association between PTSD symptoms and Aβ in whole brain and subregions, with the covariates mentioned above included. Moreover, to investigate the association between the severity of PTSD symptoms (including control, mild, moderate, and severe PTSD) and Aβ levels, an analysis of variance was performed and LSD correction was carried out to adjust for multiple comparisons. Multivariate linear regression analyses were also performed to validate the association between the severity of PTSD symptoms (mild, moderate, and severe symptoms were coded as dummy variables) and Aβ levels. The statistical analyses were performed using SPSS statistical software version 22 (IBM Corp), and the level of significance was set to a *p*-value of <0.05.

### Sensitivity analysis

To further validate the robustness of the association of PTSD symptoms with Aβ burden, logistic regression analysis was performed to explore the association between PTSD symptoms and Aβ positivity. Furthermore, the association between the severity of PTSD symptoms and Aβ positivity was explored using logistic regression analysis. In addition, to explore the effect of the sex, age, *ApoE* genotypes, and comorbidity of psychiatric diseases on PTSD and amyloid burden, the interactions sex × PTSD, age × PTSD, *ApoE* genotypes × PTSD, and psychiatric diseases × PTSD were added to the multilinear regression analysis. Pearson’s correlation analyses were used to validate the correlation between PTSD scores and regional Aβ levels, and related correlation coefficients were also calculated. Odds ratios (ORs) and 95% confidence intervals (CIs) were calculated for all regression analyses, and the level of significance was set to a *p*-value of <0.05.

## Results

**Figure 1:**
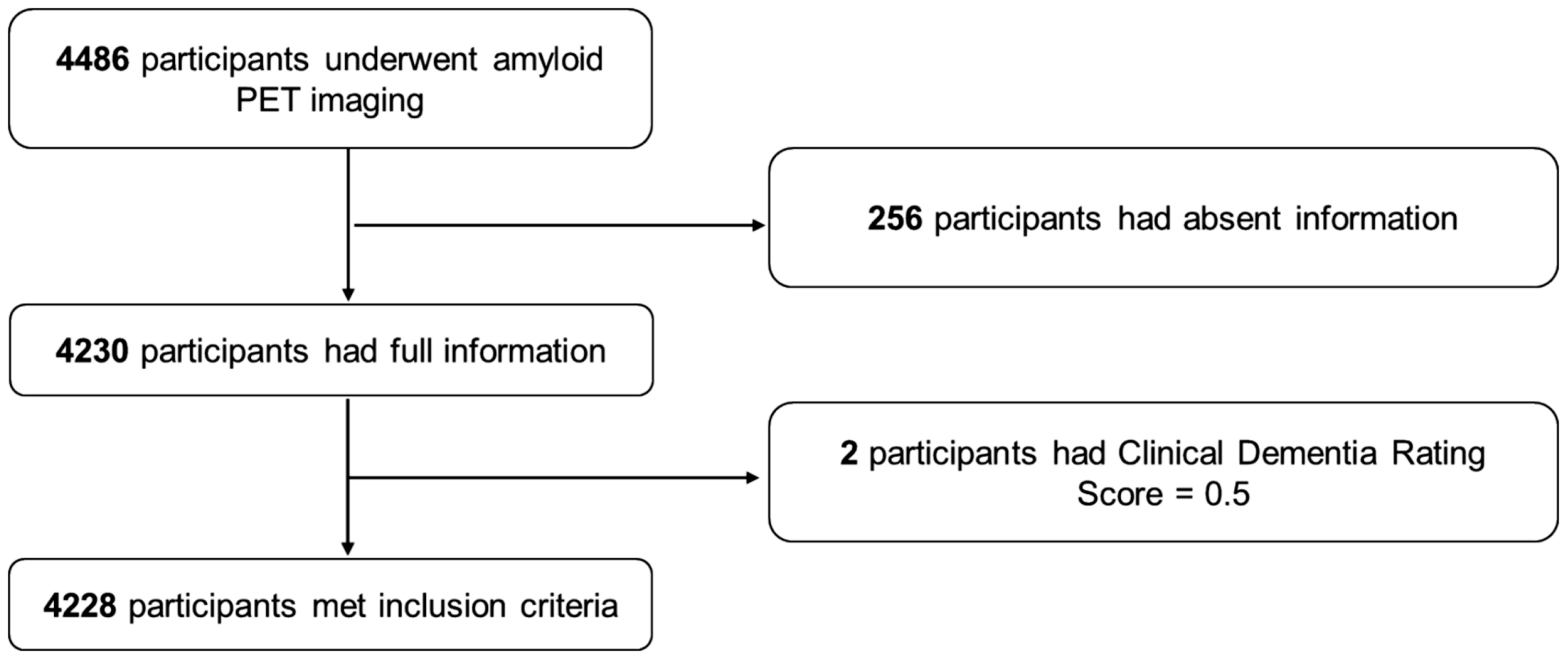
Flowchart of the process of sample inclusion.

A total of 4,486 participants underwent PET; of this sample, 4,230 had available information on caffeine consumption, demographic information, habits, *ApoE* genotypes, and comorbidities. Two participants were further excluded for preclinical dementia with a CDR of 0.5, and finally, 4,228 participants were included in the present analysis. The detailed process of sample inclusion is presented in Figure 1.

Among the included participants, 238 had PTSD symptoms and 3,990 had no PTSD symptoms. The following factors were found to be significantly different between the two groups of participants: gender, age, *ApoE* genotype, and comorbidity with psychiatric diseases. The detailed demographic and clinical characteristics of the participants, grouped by PTSD symptoms, are shown in Table 1.

**Table 1:** Demographic and clinical characteristics of the participants by posttraumatic stress disorder symptoms.

	Total	PTSD group	Control group	*p*-value
Number of observations (*N* [%])	4,228 (100.0)	238 (100.0)	3,990 (100.0)	
Sex (*N* [%])				< 0.001
Male	1723 (40.8)	57 (23.9)	1,666 (41.8)	
Female	2,505 (59.2)	181 (76.1)	2,324 (58.2)	
Age (mean [SD])	71.31 ± 4.67	70.32 ± 3.91	71.37 ± 4.71	< 0.001
BMI (mean [SD])	27.49 ± 5.10	27.04 ± 5.14	27.52 ± 5.10	0.154
Education (mean [SD])	16.59 ± 2.84	16.31 ± 2.83	16.60 ± 2.84	0.123
Marital status (*N* [%])				0.359
Married	1,239 (29.3)	76 (31.9)	1,163 (29.1)	
Not married	2,989 (70.7)	162 (68.1)	2,827 (70.9)	
Sleep duration (mean [SD])	7.10 ± 1.07	7.00 ± 1.13	7.11 ± 1.06	0.119
Alcohol drinking per day (*N* [%])				0.892
None	2,132 (50.4)	119 (50.0)	2013 (50.5)	
At least one cup	2096 (49.6)	119 (50.0)	1977 (49.5)	
Smoking per day (*N* [%])				0.321
None	4,159 (98.4)	236 (99.2)	3,923 (98.3)	
At least one package	69 (1.6)	2 (0.8)	67 (1.7)	
ApoE genotype (*N* [%])				0.013
ε22	24 (0.6)	1 (0.4)	23 (0.6)	
ε23	430 (10.2)	16 (6.7)	414 (10.4)	
ε24	109 (2.6)	8 (3.4)	101 (2.5)	
ε33	2,301 (54.4)	112 (47.1)	2,189 (54.9)	
ε34	1,230 (29.1)	92 (38.7)	1,138 (28.5)	
ε44	134 (3.2)	9 (3.8)	125 (3.1)	
Psychiatric disorders (*N* [%])				< 0.001
No	3,223 (76.2)	154 (64.7)	3,069 (76.9)	
Yes	1,005 (23.8)	84 (35.3)	921 (23.1)	
Neurological diseases (*N* [%])				0.229
No	3,261 (77.1)	176 (73.9)	3,085 (77.3)	
Yes	967 (22.9)	62 (26.1)	905 (22.7)	
Cardiovascular diseases (*N* [%])				0.953
No	1,642 (38.8)	92 (38.7)	1,550 (38.8)	
Yes	2,586 (61.2)	146 (61.3)	2,440 (61.2)	
Endocrinological diseases (*N* [%])				0.304
No	2,226 (52.6)	133 (55.9)	2,093 (52.5)	
Yes	2002 (47.4)	105 (44.1)	1897 (47.5)	

ApoE, apolipoprotein E; BMI, body mass index; PTSD, posttraumatic stress disorder.

### Relationship between PTSD symptoms and global Aβ burden

**Figure 2:**
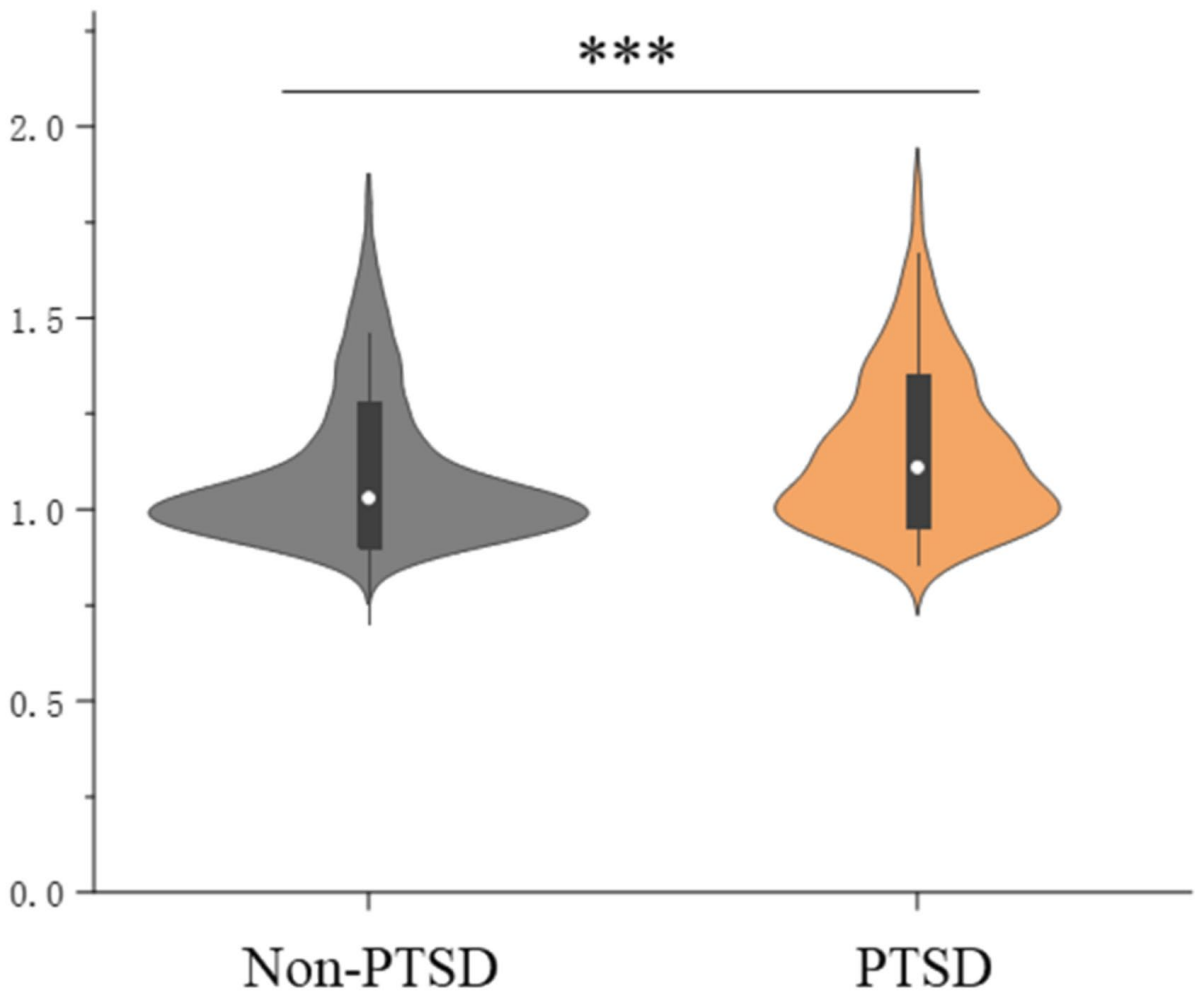
Posttraumatic stress disorder symptoms and global brain amyloid burden; ***indicates *p* < 0.001.

Multilinear linear regression showed that PTSD symptoms were significantly associated with higher whole-brain Aβ levels (1.15 ± 0.20 vs. 1.09 ± 0.19; β = 0.056; *p* < 0.001) when adjusted for covariates (Figure 2 and Table 2). Logistic regression analysis showed that PTSD symptoms were significantly associated with Aβ positivity (OR = 2.130, 95% CI = 1.601–2.834) compared to the controls (Supplementary Table S2).

**Table 2:** Multilinear regression analysis of factors associated with whole-brain amyloid burden.

Factors	Model 1		Model 2	
	β (95% CI)	*p*-value	β (95% CI)	*p*-value
Female (ref: male)	0.032	0.037	0.030	0.052
Age	0.181	<0.001	0.177	<0.001
BMI	−0.002	0.896	−0.002	0.866
Education years	−0.017	0.250	−0.017	0.250
Married (ref: no)	0.028	0.056	0.028	0.058
Sleep duration	−0.037	0.010	−0.037	0.010
Alcohol drinking (ref: no)	0.006	0.656	0.007	0.627
Smoking (ref: no)	0.005	0.747	0.004	0.773
ApoE2 (ref: no)	−0.063	<0.001	−0.065	<0.001
ApoE4 (ref: no)	0.352	<0.001	0.352	<0.001
Psychiatric diseases (ref: no)	0.039	0.008	0.042	0.005
Neurologic diseases (ref: no)	0.004	0.792	0.003	0.821
Cardiovascular diseases (ref: no)	0.013	0.392	0.012	0.400
Endocrinological diseases (ref: no)	0.011	0.466	0.011	0.457
PTSS (ref: no)	0.056	<0.001	−0.322	0.241
PTSS × Female			0.015	0.806
PTSS × Age			0.370	0.154
PTSS × ApoE2			0.008	0.614
PTSS × ApoE4			0	0.988
PTSS × Psychiatric diseases			−0.015	0.403

ApoE, apolipoprotein E; BMI, body mass index; PTSS, posttraumatic stress symptom; VIF, variance inflation factor.

Interactions, namely sex × PTSD, age × PTSD, APOE genotypes × PTSD, and psychiatric diseases × PTSD, were added to the multivariable logistic regression analysis to explore the effect of PTSD symptoms on the amyloid burden. The results did not indicate statistical significance (Table 2). These findings indicate that PTSD was significantly associated with global Aβ burden in the studied population and was influenced by sex, age, APOE genotype, or the presence of comorbid psychiatric diseases.

### Association of PTSD symptoms with regional Aβ burden

**Figure 3:**
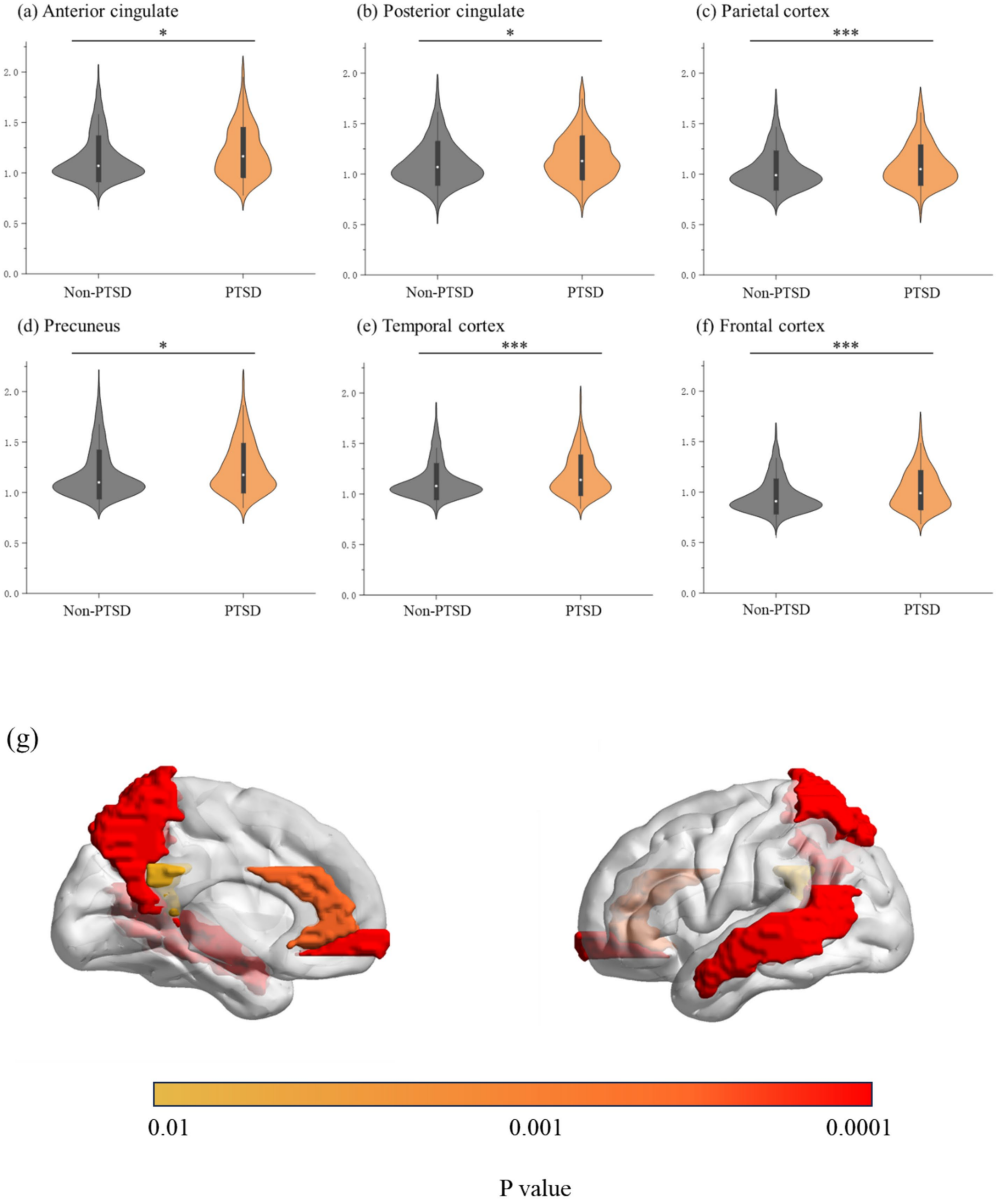
**(A–E)** Posttraumatic stress disorder symptoms and regional brain amyloid burden; **(G)** brain regions with increased amyloid burden in participants with posttraumatic stress disorder symptoms; *indicates 0.001 ≤ *p* < 0.05, and ***indicates *p* < 0.001.

In addition, significant associations were found between PTSD symptoms and regional Aβ levels (anterior cingulate: 1.21 ± 0.25 vs. 1.14 ± 0.23; β = 0.048; *p* = 0.001; posterior cingulate: 1.16 ± 0.22 vs. 1.10 ± 0.22; β = 0.040; *p* = 0.006; parietal cortex: 1.109 ± 0.21 vs. 1.04 ± 0.20; β = 0.056; *p* < 0.001; precuneus: 1.24 ± 0.25 vs. 1.18 ± 0.25; β = 0.047; *p* = 0.001; temporal cortex: 1.19 ± 0.20 vs. 1.13 ± 0.18; β = 0.061; *p* < 0.001; and frontal cortex: 1.02 ± 0.20 vs. 0.96 ± 0.18; β = 0.055; *p* < 0.001). The results are presented in Figure 3 and Supplementary Table S3. Moreover, correlation analyses further validated that PTSD symptoms were significantly correlated with regional Aβ levels (anterior cingulate: r = 0.124, *p* < 0.001; posterior cingulate: r = 0.119, *p* < 0.001; parietal cortex: r = 0.106, *p* < 0.001; precuneus: r = 0.123, *p* < 0.001; temporal cortex: r = 0.132, *p* < 0.001; and frontal cortex: r = 0.148, *p* < 0.001); the results are presented in Supplementary Figure S1.

### Association between severity of PTSD symptoms and global Aβ burden

**Figure 4:**
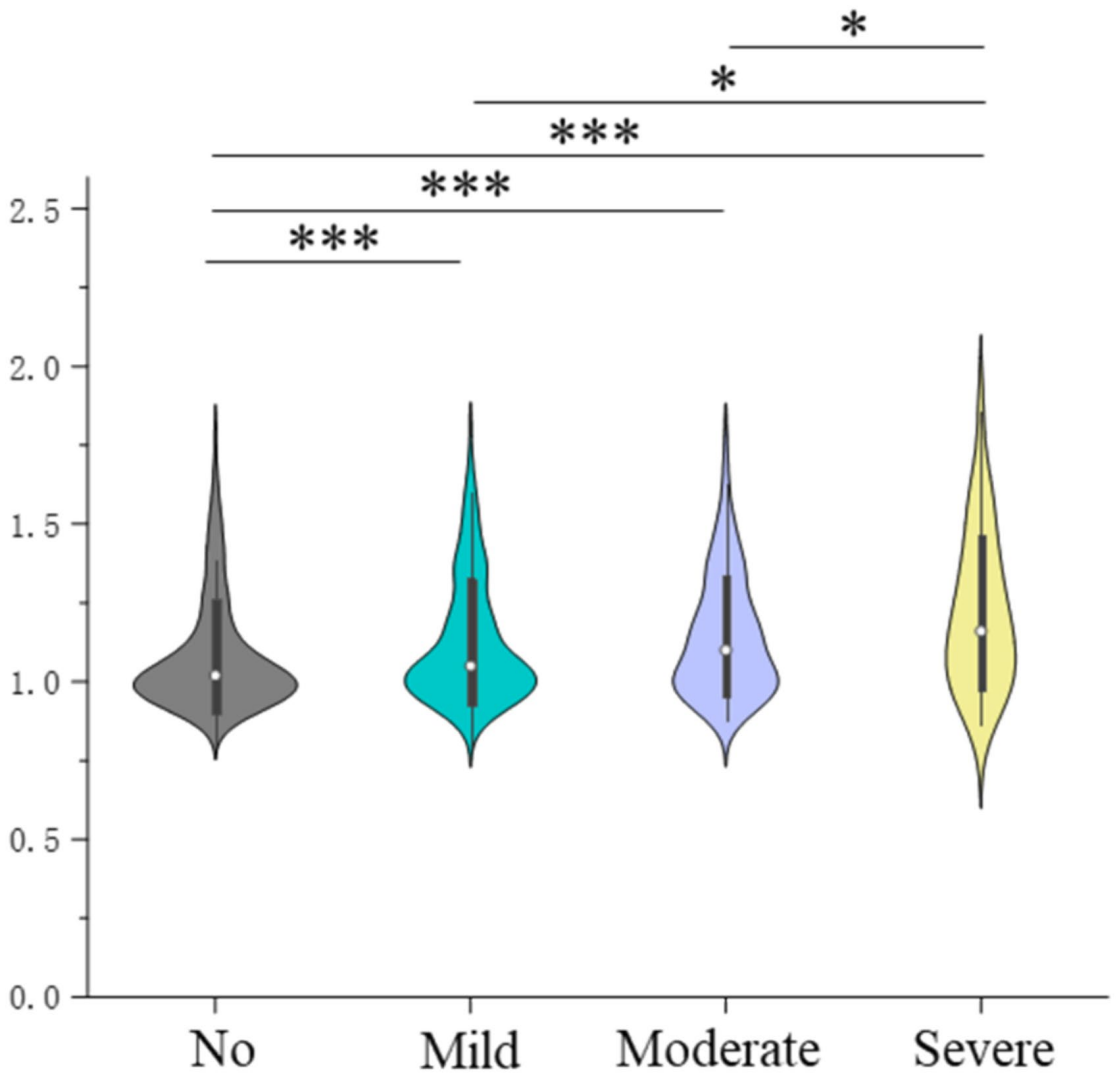
Severity of posttraumatic stress disorder symptoms and global brain amyloid burden; *indicates 0.001 ≤ *p* < 0.05, and ***indicates *p* < 0.001.

The results for the analysis of the association between the severity of PTSD symptoms and Aβ burden are presented in Figure 4 and Table 3. The Aβ burden in groups with no, mild, moderate, and severe PTSD symptoms were 1.08 ± 0.18, 1.12 ± 0.20, 1.14 ± 0.19, and 1.22 ± 0.24, respectively (*p* < 0.001). The *post-hoc* analysis showed that the group with severe PTSD symptoms had a higher global Aβ burden than the other groups, and the control group had a lower global Aβ burden than the other groups. In addition, compared to the control group, individuals with mild (OR = 1.543, 95% CI = 1.322–1.800), moderate (OR = 2.315, 95% CI = 1.700–3.153), and severe PTSD symptoms (OR = 3.709, 95% CI = 1.703–8.077) exhibited higher ORs for amyloid positivity (Supplementary Table S4).

**Table 3:** Multilinear regression analysis of the association of severity of posttraumatic stress symptoms with whole-brain amyloid burden.

Factors	β (95% CI)	*p*-value
Female (ref: male)	0.026	0.092
Age	0.183	<0.001
BMI	0.003	0.853
Education years	−0.016	0.266
Married (ref: no)	0.027	0.065
Sleep duration	−0.034	0.017
Alcohol drinking (ref: no)	0.003	0.848
Smoking (ref: no)	0.003	0.811
ApoE2 (ref: no)	−0.062	<0.001
ApoE4 (ref: no)	0.346	<0.001
Psychiatric diseases (ref: no)	0.035	0.015
Neurologic diseases (ref: no)	0.002	0.875
Cardiovascular diseases (ref: no)	0.010	0.506
Endocrinological diseases (ref: no)	0.010	0.496
Mild PTSS (ref: no)	0.079	<0.001
Moderate PTSS (ref: no)	0.054	<0.001
Severe PTSS (ref: no)	0.053	<0.001

ApoE, apolipoprotein E; BMI, body mass index; PTSS, posttraumatic stress symptom; VIF, variance inflation factor.

To further validate the association between the severity of PTSD symptoms and Aβ burden, we performed multivariable linear regression of associations between factors and Aβ burden in the whole brain and subregions, with mild, moderate, and severe symptoms as dumb variables. The results are summarized in Supplementary Table S4. The findings indicate that the global and regional Aβ burden levels are ranked in the order of severe, moderate, mild, and no PTSD symptoms, with statistical significance.

## Discussion

The current findings imply that PTSD symptoms are associated with a higher global brain Aβ burden, irrespective of sex, age, *ApoE* genotype, or comorbid psychiatric diseases. Similarly, PTSD symptoms were significantly associated with Aβ levels in all subregions, including the anterior cingulate, posterior cingulate, parietal cortex, precuneus, temporal cortex, and frontal cortex. In addition, the groups with severe PTSD symptoms exhibited higher global Aβ levels than the groups with moderate, mild, or no PTSD symptoms. The findings indicate a close relationship between PTSD and brain Aβ levels, which may provide a possible explanation for the correlation of PTSD symptoms with dementia or cognitive decline. More well-designed studies are needed to further explore the relationship of PTSD with Aβ burden and the underlying mechanisms.

The present findings also provide a neuroimaging-based explanation for the negative association between PSTD symptoms and lower ORs for dementia. As indicated in extant studies (Greenberg et al., 2014; Flatt et al., 2018), PTSD is associated with an increased risk of dementia, which may be explained by increased Aβ levels. However, the current findings may not be consistent with previous studies, which report a null association between PTSD symptoms and Aβ burden (Weiner et al., 2022; Weiner et al., 2017). These inconsistent results may be partially explained by the method used for the assessment of PTSD and the demographic information of included subjects (such as age, sex, and BMI) (Bhattarai et al., 2019; Girgenti et al., 2017). More well-designed studies are needed to explore the relationship and mechanism of PTSD symptoms and amyloid in the future.

Numerous mechanisms may help explain the relationship between PTSD symptoms and Aβ burden, for example: (1) PTSD symptoms increase peripheral and central inflammation and elevate Aβ levels. It has been suggested that inflammatory biomarkers, such as interleukin-6 (IL-6), C-reactive protein (CRP), and tumor necrosis factor (TNF), are significantly elevated in those with PTSD (Hori and Kim, 2019; Mohlenhoff et al., 2017). Chronic inflammation can disrupt the normal functioning of the immune system and exacerbate Aβ deposition. (2) Repeated exposure to stress-induced hormone dysregulation may cause the misprocessing of amyloid precursor peptides. Xie et al. (2013) showed that stress and glucocorticoids promote the misprocessing of amyloid precursor peptides in the rat hippocampus, a brain region within the temporal cortex, as well as in the prefrontal cortex, resulting in the generation of Aβ. (3) Genetic variants and epigenetic alterations related to Aβ metabolism occur in individuals with PTSD. Research has shown that exposure to traumatic stress influences the expression of epigenetic genes such as *FKBP5, BDNF, and REST*, which may directly and indirectly influence Aβ levels (Rajkumar, 2023; Guo et al., 2024). (4) Mediating factors, such as disrupted sleep and comorbid mental health issues, may also accelerate the aggregation of Aβ. One of the main manifestations of PTSD is nightmares, which would disrupt sleep. Disturbed sleep and increased wakefulness are strongly associated with increased Aβ production and decreased Aβ clearance (Zheng et al., 2024; Xie et al., 2013). It is worthwhile to elucidate these factors and the association between PTSD and Aβ levels as clearly as possible.

In this study, we also found, for the first time, that PTSD symptoms were associated with elevated regional brain Aβ levels in cognitively normal older adults. This association was observed in several brain regions, including the anterior cingulate, posterior cingulate, parietal cortex, precuneus, temporal cortex, and frontal cortex. These findings may indicate that repeated exposure may impact the whole brain rather than specific regions. As mentioned above, PTSD symptoms may cause extensive changes to the whole brain, including increased peripheral and central inflammation, induced hormone dysregulation, as well as genetic and epigenetic alterations related to Aβ metabolism (Hori and Kim, 2019; Mohlenhoff et al., 2017; Guo et al., 2024). However, the exact mechanism by which PTSD symptoms cause whole-brain Aβ increases in cognitively normal older adults still needs to be investigated and validated.

In our study, a higher level of brain Aβ burden was associated with PTSD symptom severity, which suggests the existence of a dose–response relationship. The established literature on the association of the severity of PTSD symptoms with Aβ burden is limited, and this is the first study to show that individuals with severe PTSD symptoms have a higher global Aβ burden than those with moderate, mild, or no symptoms. The findings have significant clinical meaning, suggesting the importance of early diagnosis and treatment for individuals with PTSD symptoms (Al Jowf et al., 2023). Further results from well-designed and well-conducted experiments are needed to derive robust evidence regarding the association between the severity of PTSD symptoms and Aβ levels.

The strength of this study is its large population-based sample size; however, several limitations exist. First, the assessment of PTSD symptoms was based on self-reported scales, which cannot replace diagnoses by psychiatrists. Second, this study was limited to participants aged >65 years without cognitive impairment, thereby limiting the analyses to the outcome of the emerging pathology in the absence of significant cognitive dysfunction. Third, the association between specific PTSD symptoms, such as flashbacks and nightmares, and Aβ levels was not detected. Fourth, covariates, such as the classification of multiple chronic diseases, were not clear. Chronic diseases, such as cardiovascular (Stakos et al., 2020) and neurological diseases (Vaquer-Alicea and Diamond, 2019), are associated with Aβ burden. In this study, whether the individual was diagnosed with chronic disease, the number of chronic diseases, diagnosis time, and related treatment are unknown, which may bias the results. Fifth, this is a cross-sectional study. Therefore, the associations between PTSD and amyloid burden should not necessarily be considered causal relationships.

## Conclusion

The present results indicate that PTSD symptoms are associated with higher global and regional brain Aβ burden in cognitively normal older adults. In addition, increased Aβ levels were associated with the severity of PTSD symptoms. Further studies are required to explore and validate the effects of PTSD on brain amyloid burden.

## Data Availability

The datasets presented in this study can be found in online repositories. The names of the repository/repositories and accession number(s) can be found below: The data used in this study were extracted from the Anti-Amyloid Treatment in Asymptomatic Alzheimer’s (A4)/Longitudinal Evaluation of Amyloid Risk and Neurodegeneration (LEARN) Study.
