# Supplementary material for: Associations of posttraumatic stress disorder symptoms with amyloid burden in cognitively normal older adults
Source: Front Aging Neurosci. 2024 Dec 3;16:1422862. doi: 10.3389/fnagi.2024.1422862 (PMC11649661; doi:10.3389/fnagi.2024.1422862)

**Supplementary Table 1. Summary of the questions related to this study.**

| **Questions** | **Assessment/ measures** | **Types of covariates** | **Choice** |
| --- | --- | --- | --- |
| Sex: | Questionnaire | Categorical variables | Male/Female |
| Age at screening visit: | Questionnaire | Continuous variables | / |
| Body mass index: | Physically measured | Continuous variables | / |
| Education (years): | Questionnaire | Continuous variables | / |
| Marital status | Questionnaire | Categorical variables | Married/Not married (widowed; divorced; never married; unknown) |
| Average total number of hours slept at night: | Questionnaire | Continuous variables | / |
| Average number of alcoholic drinks consumed per day: | Questionnaire | Continuous variables | / |
|  |  | Categorical variables | Yes/No |
| Average number of packs smoked per day: | Questionnaire | Continuous variables | / |
|  |  | Categorical variables | Yes/No |
| ApoE Genotype: | Genotype | Categorical variables | ε22/ε23/ε24/ε33/ε34/ε44/Not available |
| Is symptom/condition ongoing? (Psychiatric) | Questionnaire | Categorical variables | Yes/No |
| Is symptom/condition ongoing? (Neurologic (other than AD)) | Questionnaire | Categorical variables | Yes/No |
| Is symptom/condition ongoing? (Cardiovascular) | Questionnaire | Categorical variables | Yes/No |
| Is symptom/condition ongoing? (Endocrine-Metabolic) | Questionnaire | Categorical variables | Yes/No |

Abbreviation: AD, Alzheimer’s disease; ApoE, apolipoprotein E.

**Supplementary Table 2. Logistic regression analysis of factors associated with whole brain amyloid burden.**

| Factors | ORs (95% CI) | P value |
| --- | --- | --- |
| Female (ref: male) | 1.081 (0.928, 1.260) | 0.317 |
| Age | 1.082 (1.065, 1.098) | <0.001 |
| BMI | 1.002 (0.988, 1.016) | 0.761 |
| Education years | 1.007 (0.983, 1.032) | 0.569 |
| Married (ref: no) | 1.035 (0.884, 1.213) | 0.668 |
| Sleep duration | 0.932 (0.874, 0.994) | 0.032 |
| Alcohol drinking (ref: no) | 1.038 (0.902, 1.194) | 0.605 |
| Smoking (ref: no) | 1.156 (0.683, 1.956) | 0.589 |
| ApoE2 (ref: no) | 0.636 (0.510, 0.794) | <0.001 |
| ApoE4 (ref: no) | 4.719 (4.092, 5.442) | <0.001 |
| Psychiatric diseases (ref: no) | 1.077 (0.914, 1.270) | 0.377 |
| Neurologic diseases (ref: no) | 1.147 (0.975, 1.349) | 0.098 |
| Cardiovascular diseases (ref: no) | 1.039 (0.897, 1.203) | 0.613 |
| Endocrinological diseases (ref: no) | 1.101 (0.957, 1.267) | 0.177 |
| PTSS (ref: no) | 2.130 (1.601, 2.834) | <0.001 |

Abbreviations: ApoE, apolipoprotein E; BMI, body mass index; ORs: odds ratios; PTSS, posttraumatic stress symptom.

**Supplementary Table 3. Multilinear regression of factors associated with amyloid burden in different brain regions.**

(a) Anterior cingulate

| Factors | β (95% CI) | P value |
| --- | --- | --- |
| Female (ref: male) | 0.006 | 0.696 |
| Age | 0.153 | <0.001 |
| BMI | 0.017 | 0.248 |
| Education years | -0.012 | 0.400 |
| Married (ref: no) | 0.030 | 0.040 |
| Sleep duration | -0.059 | <0.001 |
| Alcohol drinking (ref: no) | 0.005 | 0.735 |
| Smoking (ref: no) | -0.003 | 0.807 |
| ApoE2 (ref: no) | -0.064 | <0.001 |
| ApoE4 (ref: no) | 0.349 | <0.001 |
| Psychiatric diseases (ref: no) | 0.032 | 0.029 |
| Neurologic diseases (ref: no) | 0.001 | 0.935 |
| Cardiovascular diseases (ref: no) | 0.007 | 0.638 |
| Endocrinological diseases (ref: no) | 0.009 | 0.538 |
| PTSS (ref: no) | 0.048 | 0.001 |

(b) Posterior cingulate

| Factors | β (95% CI) | P value |
| --- | --- | --- |
| Female (ref: male) | 0.094 | <0.001 |
| Age | 0.167 | <0.001 |
| BMI | 0.023 | 0.137 |
| Education years | -0.029 | 0.051 |
| Married (ref: no) | 0.019 | 0.217 |
| Sleep duration | -0.016 | 0.286 |
| Alcohol drinking (ref: no) | 0.007 | 0.614 |
| Smoking (ref: no) | 0.007 | 0.610 |
| ApoE2 (ref: no) | -0.035 | 0.018 |
| ApoE4 (ref: no) | 0.278 | <0.001 |
| Psychiatric diseases (ref: no) | 0.040 | 0.007 |
| Neurologic diseases (ref: no) | 0.007 | 0.622 |
| Cardiovascular diseases (ref: no) | 0.025 | 0.101 |
| Endocrinological diseases (ref: no) | 0.011 | 0.455 |
| PTSS (ref: no) | 0.040 | 0.006 |

(c) Parietal cortex

| Factors | β (95% CI) | P value |
| --- | --- | --- |
| Female (ref: male) | -0.066 | <0.001 |
| Age | 0.148 | <0.001 |
| BMI | -0.025 | 0.091 |
| Education years | -0.006 | 0.673 |
| Married (ref: no) | 0.023 | 0.130 |
| Sleep duration | -0.012 | 0.400 |
| Alcohol drinking (ref: no) | 0.003 | 0.857 |
| Smoking (ref: no) | 0.009 | 0.537 |
| ApoE2 (ref: no) | -0.067 | <0.001 |
| ApoE4 (ref: no) | 0.300 | <0.001 |
| Psychiatric diseases (ref: no) | 0.025 | 0.091 |
| Neurologic diseases (ref: no) | -0.002 | 0.909 |
| Cardiovascular diseases (ref: no) | 0.025 | 0.099 |
| Endocrinological diseases (ref: no) | 0.004 | 0.786 |
| PTSS (ref: no) | 0.056 | <0.001 |

(d) Precuneus

| Factors | β (95% CI) | P value |
| --- | --- | --- |
| Female (ref: male) | -0.012 | 0.442 |
| Age | 0.186 | <0.001 |
| BMI | 0.044 | 0.003 |
| Education years | -0.025 | 0.086 |
| Married (ref: no) | 0.029 | 0.050 |
| Sleep duration | -0.023 | 0.109 |
| Alcohol drinking (ref: no) | 0.010 | 0.504 |
| Smoking (ref: no) | 0.010 | 0.464 |
| ApoE2 (ref: no) | -0.062 | <0.001 |
| ApoE4 (ref: no) | 0.340 | <0.001 |
| Psychiatric diseases (ref: no) | 0.046 | 0.002 |
| Neurologic diseases (ref: no) | 0.011 | 0.422 |
| Cardiovascular diseases (ref: no) | 0.015 | 0.321 |
| Endocrinological diseases (ref: no) | 0.019 | 0.180 |
| PTSS (ref: no) | 0.047 | 0.001 |

(e) Temporal cortex

| Factors | β (95% CI) | P value |
| --- | --- | --- |
| Female (ref: male) | 0.060 | <0.001 |
| Age | 0.201 | <0.001 |
| BMI | -0.090 | <0.001 |
| Education years | -0.009 | 0.554 |
| Married (ref: no) | 0.023 | 0.127 |
| Sleep duration | -0.035 | 0.014 |
| Alcohol drinking (ref: no) | 0.013 | 0.358 |
| Smoking (ref: no) | 0 | 0.986 |
| ApoE2 (ref: no) | -0.060 | <0.001 |
| ApoE4 (ref: no) | 0.309 | <0.001 |
| Psychiatric diseases (ref: no) | 0.032 | 0.026 |
| Neurologic diseases (ref: no) | 0.002 | 0.902 |
| Cardiovascular diseases (ref: no) | -0.003 | 0.824 |
| Endocrinological diseases (ref: no) | 0.012 | 0.406 |
| PTSS (ref: no) | 0.061 | <0.001 |

(f) Frontal cortex

| Factors | β (95% CI) | P value |
| --- | --- | --- |
| Female (ref: male) | 0.114 | <0.001 |
| Age | 0.127 | <0.001 |
| BMI | -0.001 | 0.940 |
| Education years | -0.005 | 0.717 |
| Married (ref: no) | 0.027 | 0.070 |
| Sleep duration | -0.062 | <0.001 |
| Alcohol drinking (ref: no) | -0.002 | 0.895 |
| Smoking (ref: no) | -0.001 | 0.944 |
| ApoE2 (ref: no) | -0.059 | <0.001 |
| ApoE4 (ref: no) | 0.340 | <0.001 |
| Psychiatric diseases (ref: no) | 0.031 | 0.034 |
| Neurologic diseases (ref: no) | -0.001 | 0.945 |
| Cardiovascular diseases (ref: no) | -0.002 | 0.873 |
| Endocrinological diseases (ref: no) | -0.002 | 0.881 |
| PTSS (ref: no) | 0.055 | <0.001 |

Abbreviations: ApoE, apolipoprotein E; BMI, body mass index; PTSS, posttraumatic stress symptom.

**Supplementary Table 4. Logistic regression analysis of severity of posttraumatic stress symptom with whole brain amyloid burden.**

| Factors | ORs (95% CI) | P value |
| --- | --- | --- |
| Female (ref: male) | 1.050 (0.901, 1.225) | 0.532 |
| Age | 1.083 (1.067, 1.100) | <0.001 |
| BMI | 1.004 (0.990, 1.019) | 0.546 |
| Education years | 1.008 (0.983, 1.033) | 0.537 |
| Married (ref: no) | 1.031 (0.879, 1.209) | 0.707 |
| Sleep duration | 0.939 (0.880, 1.001) | 0.055 |
| Alcohol drinking (ref: no) | 1.018 (0.885, 1.172) | 0.799 |
| Smoking (ref: no) | 1.142 (0.675, 1.931) | 0.621 |
| ApoE2 (ref: no) | 0.640 (0.512, 0.800) | <0.001 |
| ApoE4 (ref: no) | 4.645 (4.026, 5.360) | <0.001 |
| Psychiatric diseases (ref: no) | 1.056 (0.895, 1.246) | 0.515 |
| Neurologic diseases (ref: no) | 1.138 (0.967, 1.339) | 0.120 |
| Cardiovascular diseases (ref: no) | 1.025 (0.884, 1.188) | 0.742 |
| Endocrinological diseases (ref: no) | 1.097 (0.953, 1.263) | 0.195 |
| Mild PTSS (ref: no) | 1.543 (1.322, 1.800) | <0.001 |
| Moderate PTSS (ref: no) | 2.315 (1.700, 3.153) | <0.001 |
| Severe PTSS (ref: no) | 3.709 (1.703, 8.077) | 0.001 |

Abbreviations: ApoE, apolipoprotein E; BMI, body mass index; ORs: odds ratios; PTSS, posttraumatic stress symptom.

**Supplementary Fig. 1. Correlation between posttraumatic stress symptom scores and regional brain amyloid burden.**


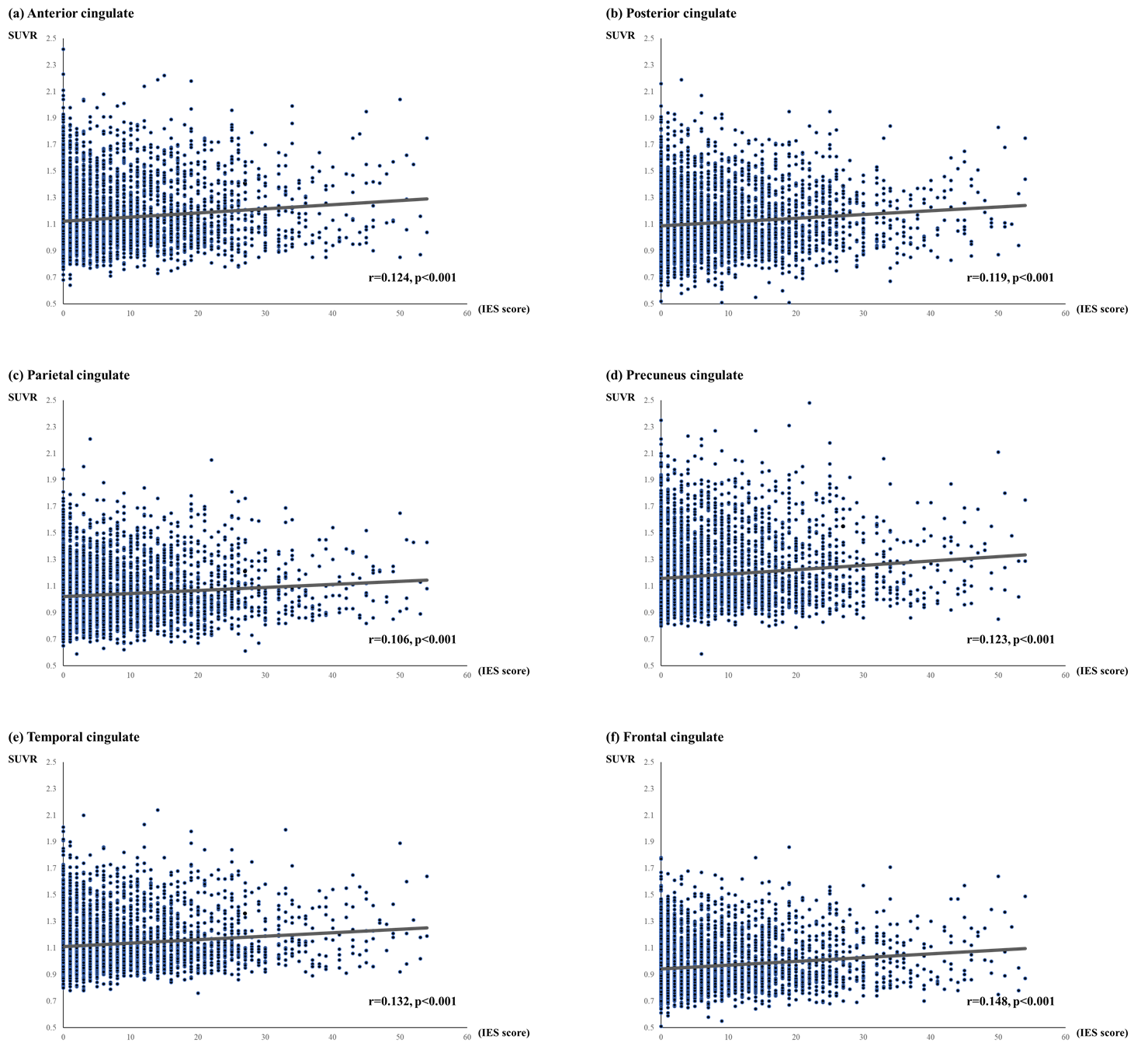

Supplement: Supplementary file 1 [file Data_Sheet_1.docx]
